# Supplementary material for: A Single-Dose Bundibugyo Virus Vaccine Protects Macaques Within 3 Days
Source: bioRxiv. 2026 Jun 15:2026.06.14.732188. Preprint. [Version 1] doi: 10.64898/2026.06.14.732188 (PMC13307998; doi:10.64898/2026.06.14.732188)
Supplement: Supplement 1 [file NIHPP2026.06.14.732188v1-supplement-1.pdf]

bioRxiv preprint doi: https://doi.org/10.64898/2026.06.14.732188; this version posted June 15, 2026. The copyright holder for this preprint (which was not certified by peer review) is the author/funder. This article is a US Government work. It is not subject to copyright under 17 USC 105 and is also made available for use under a CC0 license.

Variable	by Variable	Spearman ρ	Prob>|ρ|
Clinical Scores	ADCD	0.164889378	0.557032618
Clinical Scores	ADCP	-0.59703155	0.01878077
Clinical Scores	ADNKA CD16 MFI	0.554088776	0.032092424
Clinical Scores	ADNKA Granzyme B	-0.477957497	0.071546051
Clinical Scores	ADNP	-0.14341725	0.610111667
Clinical Scores	IgG	-0.66322172	0.007033743
Clinical Scores	NT50	-0.55850037	0.030467972
Temperature	ADCD	-0.0143	0.9596
Temperature	ADCP	-0.5293	0.0425
Temperature	ADNKA CD16 MFI	0.3841	0.1575
Temperature	ADNKA Granzyme B	-0.5338	0.0404
Temperature	ADNP	-0.0719	0.7989
Temperature	IgG	-0.5627	0.029
Temperature	NT50	-0.6593	0.0082

Supplemental Table 1. Spearman analysis of clinical outcomes and antigen-specific humoral responses 6 DPC. Spearman correlation coefficients (ρ) and two-tailed p-values highlighting relationships that are statistically significant ($p < 0.05$). No adjustments were made to the data prior to analysis.

bioRxiv preprint doi: https://doi.org/10.64898/2026.06.14.732188; this version posted June 15, 2026. The copyright holder for this preprint (which was not certified by peer review) is the author/funder. This article is a US Government work. It is not subject to copyright under 17 USC 105 and is also made available for use under a CC0 license.

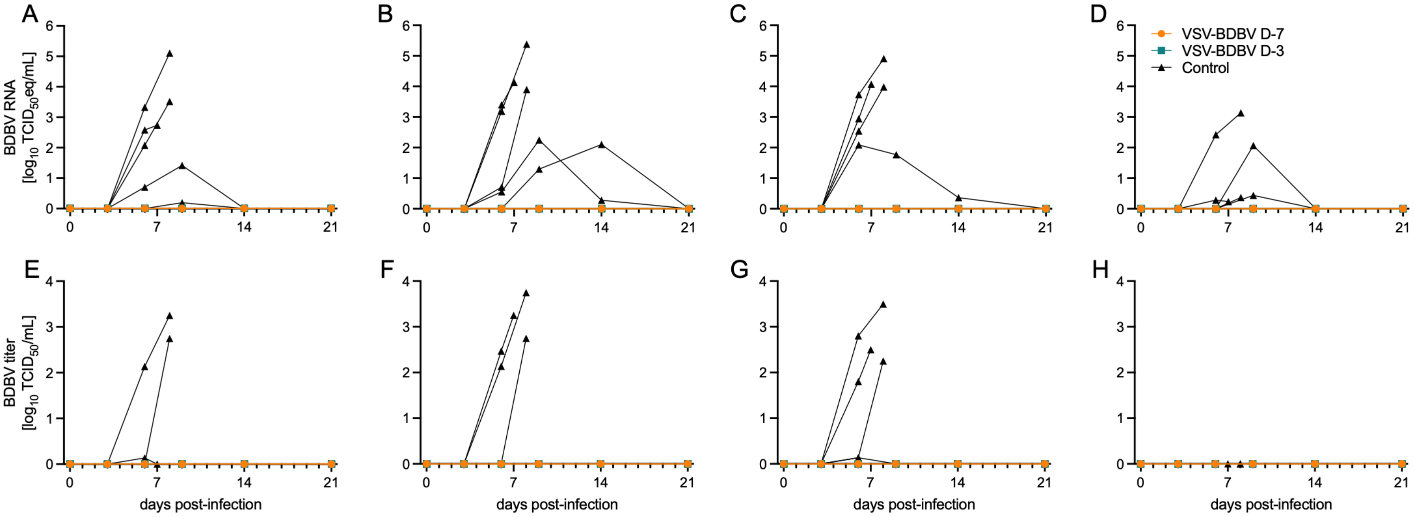

Supplemental Figure 1. Viral shedding dynamics in BDBV-infected NHPs. BDBV RNA in the (A) oral, (B) nasal, (C) rectal, and (D) urogenital swabs. BDBV titer in the (E) oral, (F) nasal, (G) rectal , and (H) urogenital swabs. $TCID_{50}$, median tissue culture infectious dose; eq, equivalent;

bioRxiv preprint doi: https://doi.org/10.64898/2026.06.14.732188; this version posted June 15, 2026. The copyright holder for this preprint (which was not certified by peer review) is the author/funder. This article is a US Government work. It is not subject to copyright under 17 USC 105 and is also made available for use under a CC0 license.

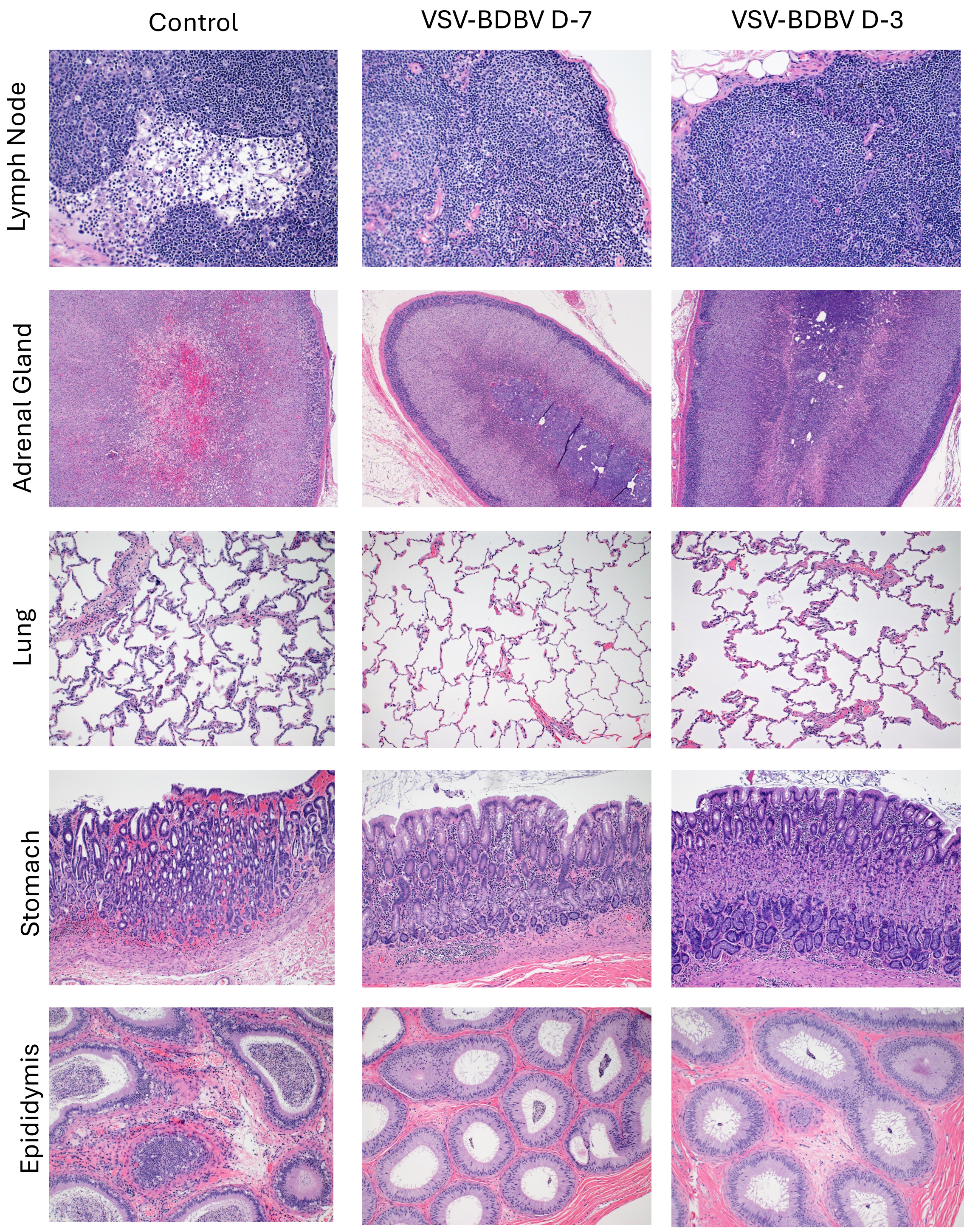

Supplemental Figure 2. Systemic histopathlogic findings in NHPs after BDBV challenge. Lymph node, adrenal gland, lung, stomach and epididymis samples collected at the time of necropsy at acute disease (control) or at study end. Hematoxylin and eosin staining imaged at 200x.

bioRxiv preprint doi: https://doi.org/10.64898/2026.06.14.732188; this version posted June 15, 2026. The copyright holder for this preprint (which was not certified by peer review) is the author/funder. This article is a US Government work. It is not subject to copyright under 17 USC 105 and is also made available for use under a CC0 license.

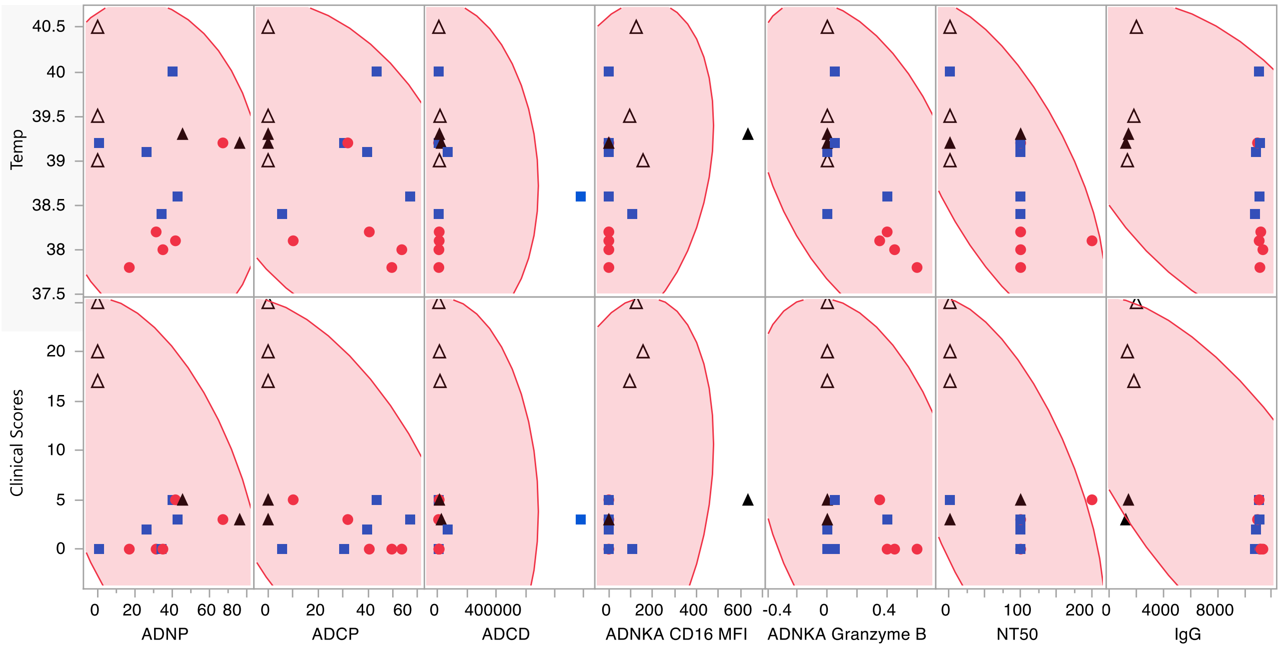

Supplemental Figure 3. Correlation analysis of survival outcomes and antigen-specific humoral responses. Scatterplot matrix showing relationship between clinical symptoms (body temperature and clinical score) and immunogenicity readouts measured on 6 days post-challenge (n=5 per group). Data sets (provided in Supplemental Table s) were analyzed using Spearman's test (two-tailed).

bioRxiv preprint doi: https://doi.org/10.64898/2026.06.14.732188; this version posted June 15, 2026. The copyright holder for this preprint (which was not certified by peer review) is the author/funder. This article is a US Government work. It is not subject to copyright under 17 USC 105 and is also made available for use under a CC0 license.

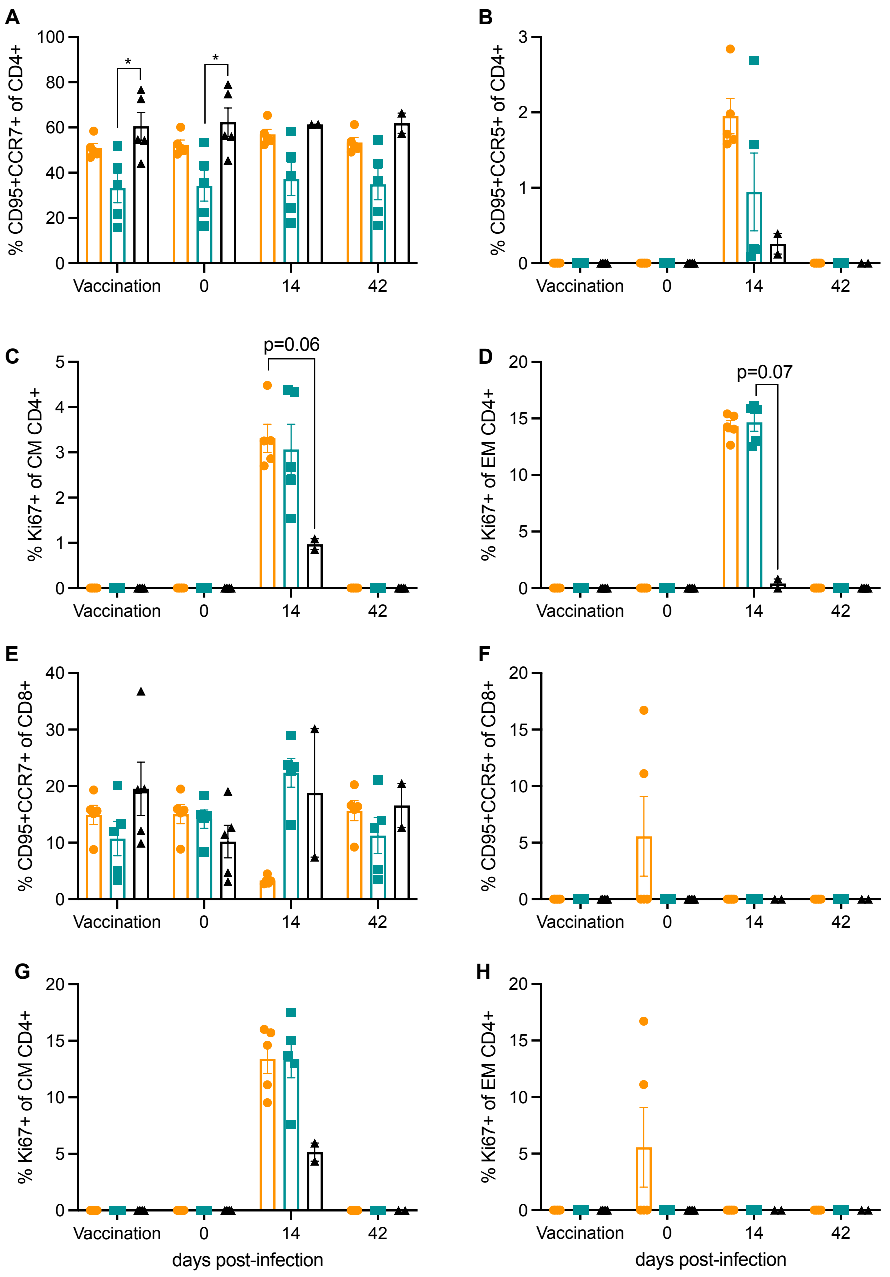

Supplemental Figure 4. Cellular immune response after vaccination and BDBV challenge. (A-D) CD4 T cells and (E-H) CD8 T cells were characterized for memory populations and activation markers were determined over time. (A) Central memory (CM) CD4 T cells; (B) Effector memory (EM) CD4 T cells; (C-D) activation of CM and EM with Ki67 staining. (E) Central memory (CM) CD8 T cells (F); effector memory (EM) CD8 T cells; (G-H) activation of CM and EM with Ki67 staining. Mean (SE) are depicted. Data sets were analyzed using Kruskal-Wallis test with Dunn's multiple comparisons, statistically significant differences are indicated as p < 0.05 (*).

bioRxiv preprint doi: https://doi.org/10.64898/2026.06.14.732188; this version posted June 15, 2026. The copyright holder for this preprint (which was not certified by peer review) is the author/funder. This article is a US Government work. It is not subject to copyright under 17 USC 105 and is also made available for use under a CC0 license.

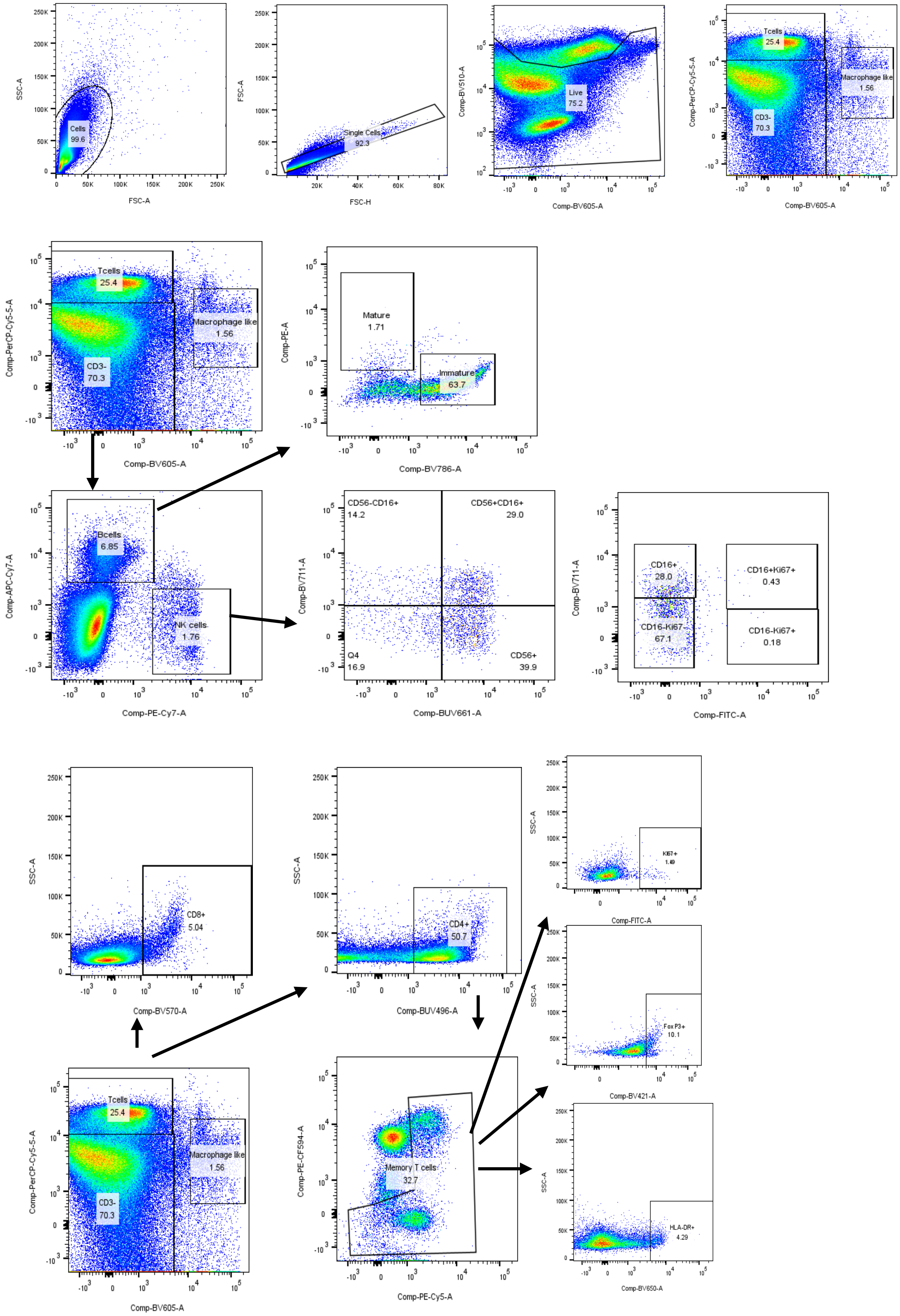

Supplemental Figure 5. Flow cytometry gating strategies.
